# Sex differences in Speech and High-frequency Hearing Loss Association With Cognitive Impairment Among Older Adults

**DOI:** 10.1093/geroni/igab046.2659

**Published:** 2021-12-17

**Authors:** Wang Jingru, Yu Ying, Guo Qi

**Affiliations:** Shanghai University of Medicine and Health Sciences, Shanghai, Shanghai, China (People's Republic)

## Abstract

Objectives: The purpose of this study was to investigate the relationship between speech-frequency hearing loss (SFHL), high-frequency hearing loss (HFHL), and cognitive impairment (CI). Then to determine whether there are any differences in gender among older community dwellers in China. Methods: This study involved 1,012 adults aged ≥60 years (428 male; average age, 72.61±5.51 years). The participants had their hearing and cognition measured using pure tone audiometry and Mini Mental State Examination (MMSE), respectively. We used the audiometric definition of hearing loss (HL) adopted by the World Health Organization (WHO). Speech-frequencies were measured as 0.5 kHz, 1 kHz, 2 kHz, and 4 kHz; high-frequencies were measured as 4 kHz and 8 kHz. Pure tone average (PTA) was measured as hearing sensitivity. Results: Our studies demonstrated a 37.6% prevalence of HL in males and a 36.0% prevalence of HL in females. Adjusted for confounding variables, the results from a multivariate analysis showed that SFHL was associated with CI in females (OR=2.400, 95% Confidence Interval=1.313–4.385) and males (OR=2.189, 95% Confidence Interval=0.599–2.944). However, HFHL was associated with CI only in females (OR=2.943, 95% Confidence Interval=1.505–5.754). HL was associated with poorer cognitive scores (P<0.05). “Registration” (P<0.05) in MMSE was associated with speech and high-frequency hearing sensitivity. Conclusion: The associations between HL and CI varied according to gender in older community-dwellers, suggesting that different mechanisms are involved in the etiology of HL. Moreover, hearing sensitivity was negatively associated with cognition scores; therefore, early screening for HL and CI among older community-dwelling adults is advised.

